# The Effects of Magnesium Ions on the Enzymatic Synthesis of Ligand-Bearing Artificial DNA by Template-Independent Polymerase

**DOI:** 10.3390/ijms17060906

**Published:** 2016-06-08

**Authors:** Yusuke Takezawa, Teruki Kobayashi, Mitsuhiko Shionoya

**Affiliations:** Department of Chemistry, Graduate School of Science, The University of Tokyo, 7-3-1 Hongo, Bunkyo-ku, Tokyo 113-0033, Japan; takezawa@chem.s.u-tokyo.ac.jp (Y.T.); tkobayashi@chem.s.u-tokyo.ac.jp (T.K.)

**Keywords:** artificial DNA, metal-mediated base pair, enzymatic synthesis, DNA polymerase, bioinorganic chemistry

## Abstract

A metal-mediated base pair, composed of two ligand-bearing nucleotides and a bridging metal ion, is one of the most promising components for developing DNA-based functional molecules. We have recently reported an enzymatic method to synthesize hydroxypyridone (**H**)-type ligand-bearing artificial DNA strands. Terminal deoxynucleotidyl transferase (TdT), a template-independent DNA polymerase, was found to oligomerize **H** nucleotides to afford ligand-bearing DNAs, which were subsequently hybridized through copper-mediated base pairing (**H**–Cu^II^–**H**). In this study, we investigated the effects of a metal cofactor, Mg^II^ ion, on the TdT-catalyzed polymerization of **H** nucleotides. At a high Mg^II^ concentration (10 mM), the reaction was halted after several **H** nucleotides were appended. In contrast, at lower Mg^II^ concentrations, **H** nucleotides were further appended to the **H**-tailed product to afford longer ligand-bearing DNA strands. An electrophoresis mobility shift assay revealed that the binding affinity of TdT to the **H**-tailed DNAs depends on the Mg^II^ concentration. In the presence of excess Mg^II^ ions, TdT did not bind to the **H**-tailed strands; thus, further elongation was impeded. This is possibly because the interaction with Mg^II^ ions caused folding of the **H**-tailed strands into unfavorable secondary structures. This finding provides an insight into the enzymatic synthesis of longer ligand-bearing DNA strands.

## 1. Introduction

DNA is a molecule that has the outstanding molecular recognition ability, through which each strand hybridizes with its counterpart in a sequence-specific manner. An adenine base (A) on one strand and a thymine base (T) on the other strand form a base pair, and so do a guanine (G) and a cytosine (C). This complementary base pairing is achieved by hydrogen bonding between the nucleobases. Owing to the high molecular recognition ability and programmability, DNA self-assembly has been extensively utilized to nanostructures and nanomaterials [1,2,3].

Over recent decades, a sustained effort has been devoted to the development of artificial metal-mediated base pairs, which are formed through metal coordination bonding instead of hydrogen bonding [4,5]. Metal coordination is one of the most employed interactions for designing the self-assembly of molecules [6] and thus has been also utilized for the construction of DNA-based materials [7,8,9]. Metallo-base pairs, consisting of two ligand-bearing nucleosides and a bridging metal ion, have unique and fascinating characteristics as noted below. (i) Metal-mediated base pairing leads to a significant thermal stabilization of DNA duplexes; (ii) the formation of the base pairs can be regulated by the addition and the removal of specific metal ions; (iii) the consecutive incorporation of metallo-base pairs provides multi-metal assembly within DNA helical structures. Due to its versatility, metal-mediated base pairing systems have attracted increasing attention in the field of nucleic acid chemistry, especially in DNA-based materials science and nanotechnology [10,11].

We have so far developed several metal-mediated artificial base pairs [12,13,14,15,16] including Cu^II^-mediated hydroxypyridone self-base pair (**H**–Cu^II^–**H**). The **H**–Cu^II^–**H** base pair is composed of two bidentate hydoxypyridone-bearing nucleotides (**H**) and a Cu^II^ ion [14]. It has been applied for the Cu^II^-dependent thermal stabilization of DNA duplexes [14] and for the regulation of the electroconductivity of DNA devices [17]. Multi-incorporation of the **H**–Cu^II^–**H** pairs yielded one-dimensional arrays of Cu^II^ ions inside DNA duplexes, which exhibited magnetic interactions [18]. In a similar manner, the discrete assembly of other metal ions, such as Mn^III^ [19], Ni^II^ [20], Ag^I^ [21,22], and Gd^III^ [15] ions, as well as heterogeneous metal assembly [23] have been constructed by utilizing a variety of ligand-bearing nucleosides.

**Scheme 1:**
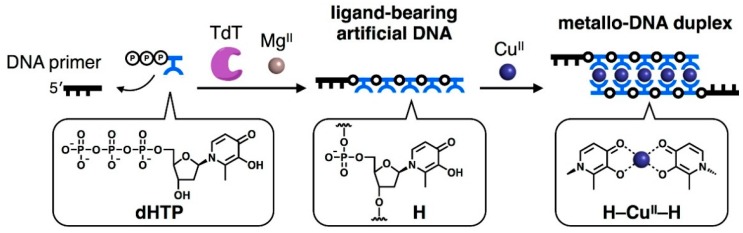
Schematic representation of polymerase synthesis of ligand-bearing artificial DNAs. The resulting DNA oligomers form metallo-DNA duplex structures through the formation of Cu^II^-mediated artificial base pairs (**H**–Cu^II^–**H**). **H**: hydroxypyridone-bearing nucleoside, d**H**TP: hydroxypyridone-bearing nucleoside triphosphate, TdT: terminal deoxynucleotidyl transferase.

In these earlier studies, ligand-bearing nucleotides were incorporated into DNA strands based on the standard solid-phase phosphoramidite chemistry. We have recently developed a method to synthesize artificial ligand-bearing DNA strands via an enzymatic polymerization reaction [24]. Enzymatic DNA synthesis is widely utilized in chemical biology as well as in DNA-based materials science [25]. Thus, polymerase synthesis of ligand-bearing artificial DNAs is expected to greatly expand the future applications of the metal-mediated base pairing system. The synthetic scheme we have developed is depicted in Scheme 1. The triphosphate derivative of the hydroxypyridone-bearing nucleoside (d**H**TP) is oligomerized to afford ligand-bearing artificial DNA strands, which are subsequently subjected to metal-mediated base pairing. We utilized a template-independent DNA polymerase, terminal deoxynucleotidyl transferase (TdT) [26,27,28], because it is known to accept not only natural deoxynucleoside triphosphates (dNTPs) but also modified and unnatural nucleoside triphosphates [29,30,31,32]. We found that TdT catalyzed the polymerization of d**H**TP, and DNA primers were successfully tailed with several **H** nucleotides. The resulting **H**-tailed strands formed metallo-DNA duplex structures through the formation of **H**–Cu^II^–**H** base pairs, which was confirmed by ultraviolet-visible (UV) spectroscopy and polyacrylamide gel electrophoresis (PAGE) [24].

However, the reaction rate of the d**H**TP incorporation by TdT was significantly lower than those of natural triphosphates (dNTPs). In addition, the polymerization seemed to be halted after about five **H** nucleotides were incorporated, even though a substantial amount of d**H**TP still remained [24]. TdT polymerase requires divalent metal ions such as Mg^II^ ions to catalyze the incorporation of nucleotide substrates [26,27,28], as is the case for template-dependent DNA polymerases. We suspect that the TdT-catalyzed polymerization was significantly affected by the interaction between the ligand-bearing **H** nucleotide and coexistent Mg^II^ ions. In this study, we investigated effects of Mg^II^ ions on the TdT-catalyzed synthesis of hydroxypyridone-type ligand-bearing artificial DNAs. Furthermore, a possible reason for the arrest in polymerization will be discussed in terms of the interaction between Mg^II^ ions and the product, namely **H**-tailed DNA oligomers.

## 2. Results and Discussion

### 2.1. Interaction of Mg^II^ Ions with Hydroxypyridone-Bearing Nucleotides (**H**)

**Figure 1:**
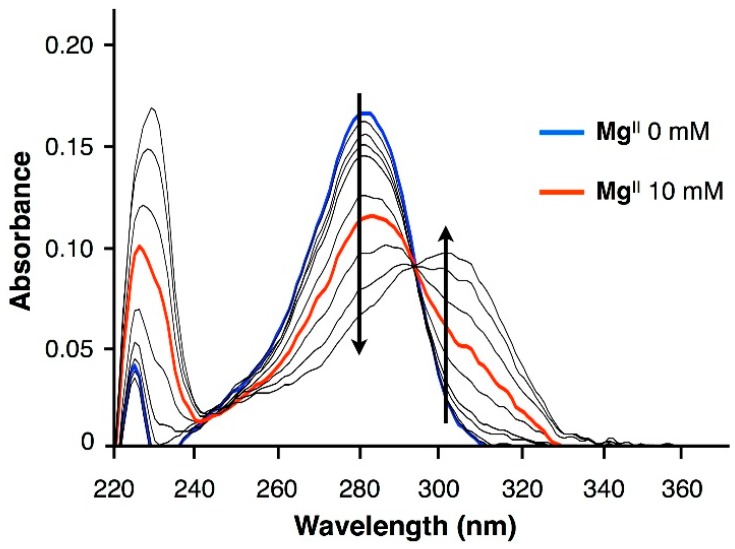
Ultraviolet-visible (UV) spectral changes of hydroxypyridone-bearing nucleoside triphosphate (d**H**TP) at various concentrations of Mg^II^ ions. d**H**TP: 100 µM, Mg^II^: 0, 0.1, 0.5, 1.0, 2.0, 5.0, 10, 20, 50, and 100 mM, in 25 mM MOPS (3-(*N*-morpholino)propanesulfonic acid) buffer (pH = 7.0), *l* = 0.1 cm, at room temperature. Blue line: 0 mM, red line: 10 mM, black lines: 0.1, 0.5, 1.0, 2.0, 5.0, 20, 50, and 100 mM. Arrows indicate the changes in the absorbance upon increasing the Mg^II^ concentration.

Hydroxypyridone ligand is known as a potent chelator of transition metal ions such as Cu^II^ [33] and Fe^III^ [34,35,36]. Although the Irving–Williams series suggests that Mg^II^ ions, compared with Fe^III^ and Cu^II^, have weaker binding affinity to hydroxypyridone, there are nonnegligible interactions between hydroxypyridone-bearing nucleotide (**H**) and highly concentrated Mg^II^ ions, as shown in Figure 1. The UV spectra of hydroxypyridone-bearing nucleoside triphosphate (d**H**TP) were measured in the presence of various concentrations of Mg^II^ ions (0–100 mM) under a neutral condition (pH = 7.0). In the absence of Mg^II^ ions, the UV spectrum has an absorption maximum at 282 nm (blue line). With increasing Mg^II^ concentrations, the absorption around 282 nm gradually decreased, and a new absorption band appeared around 303 nm. The appearance of the new band is indicative of the deprotonation of the 3-hydroxyl group of the hydroxypyridone moiety. This spectral change with an isosbestic point at 294 nm is very similar to that observed for the complexation of hydroxypyridone-bearing nucleoside (**H**) and Cu^II^ ions [14]. Thus, the result strongly indicates that Mg^II^ ions bound to the hydroxypyridone ligand to some extent. In the presence of 10 mM of Mg^II^ ions in accordance with the TdT-catalyzed polymerization, the absorption at 282 nm was reduced to two-thirds, and the absorption at 303 nm increased threefold (red line). These results suggest that a significant amount of hydroxypyridone could interact with Mg^II^ ions at high concentrations. Thus, it is likely that the Mg^II^ concentration affects the TdT-catalyzed polymerization of d**H**TP.

### 2.2. Effects of Mg^II^ Ions on the Terminal Deoxynucleotidyl Transferase (TdT)-Catalyzed Polymerization of d**H**TP

**Figure 2:**
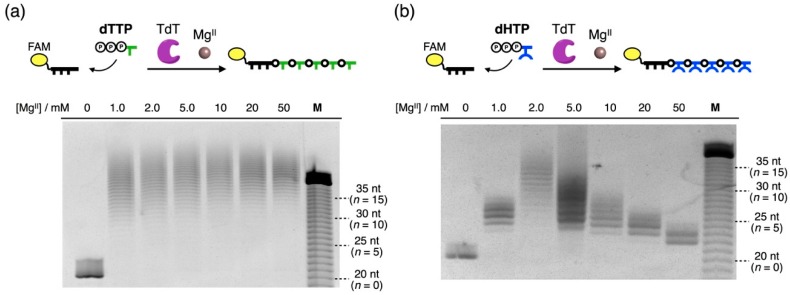
Effects of Mg^II^ concentrations on the TdT-catalyzed polymerization of (**a**) natural thymidine triphosphate (dTTP) and (**b**) artificial d**H**TP. Primer (5′-FAM-dT_20_-3′): 5.0 µM, dTTP or d**H**TP: 100 µM (20 equivalents), TdT: 2 U/µL, MgCl_2_: 0, 1.0, 2.0, 5.0, 10, 20, and 50 mM, 37 °C, 24 h. The products were analyzed by denaturing polyacrylamide gel electrophoresis (PAGE). The bands were detected by fluorescence of the fluorescein (FAM) moiety. “*n*” represents the number of the appended nucleotides. “nt” is short for “nucleotide”.

Next, we investigated the effects of Mg^II^ ions on the enzymatic reaction by varying the concentrations of Mg^II^ ions (0 to 50 mM) in the reaction buffer. A fluorescein (FAM)-labeled DNA primer (5′-FAM-dT_20_-3′, 5.0 µM) was incubated with 20 equivalents of natural thymidine triphosphate (dTTP) or artificial hydroxypyridone-bearing nucleoside triphosphate (d**H**TP) in the presence of TdT (2 U/µL). After 24-h incubation at 37 °C, the reaction products were analyzed by denaturing polyacrylamide gel electrophoresis (PAGE) (Figure 2). In the absence of Mg^II^ ions, only the primer strand was observed on the gel, showing that neither dTTP nor d**H**TP was appended to the primer by the enzyme. This result corroborated the fact that TdT requires Mg^II^ ions as a cofactor for its enzymatic activity [26,27,28].

In the range of 1.0–50 mM Mg^II^ ions, characteristic ladder patterns were observed on the gel. This result suggests that TdT catalyzed the sequential elongation of DNA oligomers. In the reaction with dTTP, TdT appended on average 20 dTTPs to the primer regardless of the Mg^II^ concentrations. In contrast, the lengths of the polymerization products were highly dependent on the Mg^II^ concentrations when d**H**TP was used as a substrate. Under the original reaction condition (*i.e.*, 10 mM Mg^II^), *ca.* 5.5 **H** nucleotides were polymerized on average. In the presence of a large amount of Mg^II^ ions, TdT polymerized fewer d**H**TPs. With decreasing Mg^II^ concentrations, further elongated products were obtained. The most efficient elongation was observed in the presence of 2.0 mM of Mg^II^ ions. These results raised the possibility that the enzymatic reaction was partially inhibited by excessive Mg^II^ ions.

**Figure 3:**
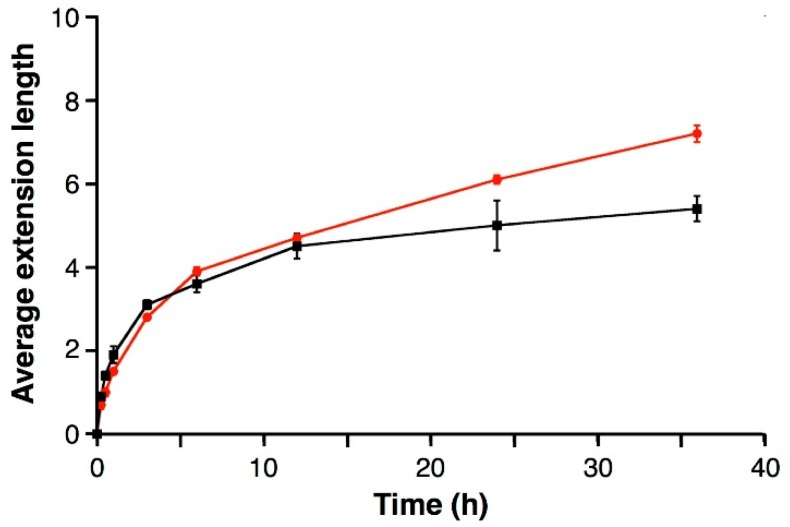
Time-course analysis of the TdT-catalyzed polymerization of d**H**TP in the presence of 2.0 mM (red circles) and 10 mM (black squares) of Mg^II^ ions. Primer (5′-FAM-dT_20_-3′): 5.0 µM, d**H**TP: 100 µM (20 equivalents), TdT: 2 U/µL, MgCl_2_: 2.0 or 10 mM, 37 °C. Average extension length was estimated based on the intensity of the bands on the gel. Averages of at least three independent experiments are shown in the figure. Error bars indicate the standard errors.

The time-course analysis of the polymerase reaction was performed in the presence of 2.0 mM of Mg^II^ (Figure 3, red line) and 10 mM of Mg^II^ ions (black line). Under the original condition (10 mM Mg^II^), the reaction slowed down significantly after 12 h when 4–5 **H** nucleotides were appended to the 3′ terminus of the primer. We have previously speculated that TdT enzyme had a low affinity to the extended products tailed with unnatural **H** nucleotides and thus failed in further incorporation of the triphosphates [24]. In the presence of 2.0 mM of Mg^II^ ions, the reaction initially proceeded at a comparable rate to the case with 10 mM of Mg^II^. Interestingly, the polymerization still continued even after 4–5 **H** nucleotides were appended. As a result, longer ligand-bearing DNA oligomers were obtained at the lower Mg^II^ concentration. These results clearly show that the Mg^II^ concentration had a significant influence on the further addition of **H** nucleotides to the **H**-tailed oligonucleotides (*i.e.*, 5′-FAM-dT_20_**H***_n_*-3′).

**Figure 4:**
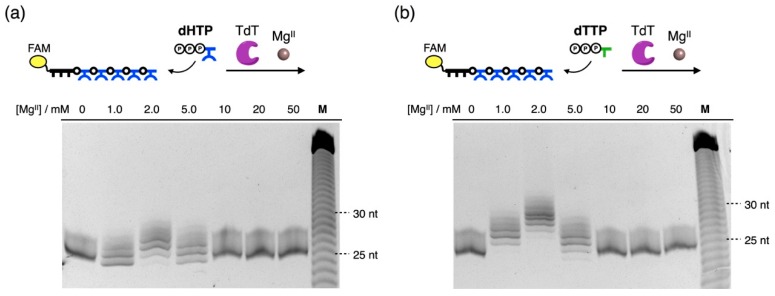
TdT-catalyzed elongation of ligand-bearing artificial DNA strands with (**a**) d**H**TP and (**b**) dTTP. Primer (5′-FAM-dT_20_**H**_5_-3′): 5.0 µM, dTTP or d**H**TP: 100 µM (20 equivalents), TdT: 2 U/µL, MgCl_2_: 0, 1.0, 2.0, 5.0, 10, 20, and 50 mM, 37 °C, 24 h. The products were analyzed by denaturing PAGE. The bands were detected by fluorescence of the fluorescein (FAM) moiety. “nt” is short for “nucleotide”.

In order to gain further insight on the effects of Mg^II^ ions, we performed the polymerization reaction using an **H**-tailed oligonucleotide (5′-FAM-dT_20_**H**_5_-3′) as a primer. Figure 4a shows the result of the polymerization of d**H**TP at various concentrations of Mg^II^ ions. While primer extension was hardly observed in the range of 10–50 mM Mg^II^, the incorporation of d**H**TP occurred at lower concentrations of Mg^II^ ions. Indeed, when the Mg^II^ concentration was 2.0 mM, the most extended product was obtained. The same primer extension experiments were carried out with a natural dTTP in place of d**H**TP (Figure 4b). The polymerization did not proceed at higher concentrations of Mg^II^ ions, and the most efficient elongation was observed at 2.0 mM Mg^II^. Thus, incorporation efficiencies of both d**H**TP and dTTP exhibited high and similar dependency on the Mg^II^ concentrations. Consequently, Mg^II^ ions are more likely to affect the binding affinity between TdT and the **H**-tailed primer strand rather than recognition of the d**H**TP substrate.

### 2.3. Effects of Mg^II^ Ions on the Affinity between TdT and **H**-Tailed Strands

**Figure 5:**
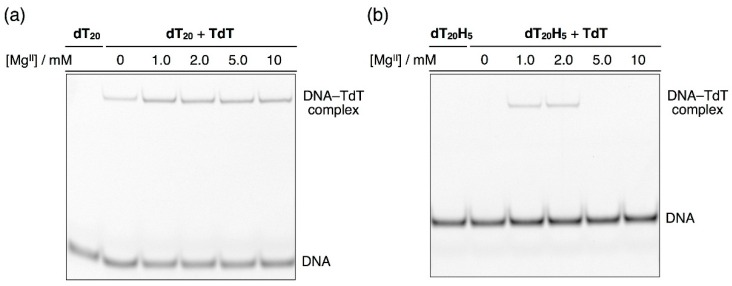
Effects of Mg^II^ concentrations on the binding of TdT with (**a**) natural DNA primers (5’-FAM-dT_20_-3′) and (**b**) artificial ligand-bearing DNA primers (5′-FAM-dT_20_**H**_5_-3′). EMSA was performed using an EDTA-free buffer at 10–15 °C. 5′-FAM-dT_20_-3′ or 5′-FAM-dT_20_**H**_5_-3′: 5.0 µM, TdT: 2 U/µL, MgCl_2_: 0, 1.0, 2.0, 5.0 and 10 mM.

We hypothesized that the Mg^II^-dependent nature of the d**H**TP polymerization stems from the binding affinity of the TdT polymerase to the **H**-tailed DNA strands. In order to elucidate the binding affinity of TdT, an electrophoresis mobility shift assay (EMSA) was conducted with a natural DNA primer 5′-FAM-dT_20_-3′ and an **H**-tailed primer 5′-FAM-dT_20_**H**_5_-3′ (Figure 5). A binary complex of the natural DNA primer and TdT was observed on the gel regardless of the Mg^II^ concentration (Figure 5a). In contrast, the binding affinity to the **H**-tailed DNA was dependent on the Mg^II^ concentration (Figure 5b). With 5.0–10 mM of Mg^II^ ions, TdT hardly bound to the artificial DNA strand, which accounts for why the TdT-catalyzed reaction halted after several **H** nucleotides were appended. At lower concentrations of Mg^II^ ions (1.0–2.0 mM), the binding of TdT to the **H**-tailed DNA strand was distinctly detected. This result indicates that the TdT polymerase recognized the **H**-tailed products as a primer for further extension reaction.

TdT was found to require DNA primers consisting of at least three nucleotides to initiate the polymerization reaction [37,38]. X-ray studies also evidenced that TdT tightly accommodates at least three nucleotides at the 3′ terminus of the primer [39]. These findings suggest that the structure of the 3′ end of the primer affects the molecular recognition by TdT polymerase. In fact, TdT cannot catalyze the elongation of primers tailed with ribonucleotides, although it can accept ribonucleoside trihospahtes (rNTPs) as substrates. This was previously ascribed to the unfavorable conformation of the single-stranded RNA strand [40,41]. The UV-based titration experiment shown in Figure 1 indicates that the **H** ligands on the **H**-tailed DNA strands interact with Mg^II^ ions. Thus, it is likely that the 3′ terminus of the **H**-tailed strands is folded into a certain unfavorable secondary structure at higher Mg^II^ concentrations. As a result, TdT failed to bind to the **H**-tailed DNA strands; thus, further polymerization was impeded.

## 3. Materials and Methods

### 3.1. Materials and General Procedures

A natural DNA primer strand labeled with 6-carboxyfluorescein (5′-FAM-dT_20_-3′) was purchased from Japan Bio Service Co., Ltd. (Saitama, Japan) at HPLC purification grade. Hydroxypyridone-bearing nucleoside triphosphate (d**H**TP) was synthesized according to a previous report [24]. Terminal deoxynucleotidyl transferase (TdT) was purchased from New England Biolabs, Inc. (Ipswich, Massachusetts, USA). The FAM-labeled single-stranded DNA ladder marker was prepared by the treatment of a FAM-labeled T_38_ oligomer with DNase I (Promega (Fitchburg, WI, USA)). An **H**-tailed DNA primer, 5′-FAM-dT_20_**H**_5_-3′, was synthesized by the TdT-catalyzed primer extension using d**H**TP as a substrate and purified by denaturing PAGE and isopropanol precipitation in a manner similar to the previous report [24]. The concentration of the DNA strands was determined based on the absorbance of the FAM moiety (λ = 495 nm, ε_495_ = 3.75 × 10^4^ M^−^^1^·cm^−^^1^ at pH 7.0). Denaturing polyacrylamide gel electrophoresis (PAGE) was carried out with 21% polyacrylamide gel with 7 M urea. The gels were analyzed using Alpha imager mini (LMS Co., Ltd. (Tokyo, Japan)) and a blue-LED (470 nm) transilluminator (Optocode Corp. (Tokyo, Japan)). The bands were detected by FAM fluorescence, and their intensities were quantified using ImageJ (National Institute of Health (NIH), Bethesda, MD, USA).

### 3.2. Ultraviolet-Visible (UV) Spectroscopy

Hydroxypyridone-bearing nucleoside triphosphate (d**H**TP) was combined with various concentrations of MgCl_2_ (0, 0.1, 0.5, 1.0, 2.0, 5.0, 10, 20, 50, and 100 mM) in a MOPS (3-(*N*-morpholino)propanesulfonic acid) buffer (25 mM, pH = 7.0). After being incubated at 25 °C for 1 h, the samples were subjected to the UV measurement at room temperature using a NanoDrop 2000 spectrometer (Thermo Scientific (Waltham, MA, USA)) with a path length of 0.1 cm.

### 3.3. Primer Extension Experiments

A FAM-labeled DNA primer was combined with a nucleoside triphosphate on ice in a reaction buffer (20 mM of Tris-acetate (pH 7.9), 50 mM of KOAc) containing various concentrations of Mg^II^ ions (e.g., 10 mM of Mg(OAc)_2_). After the addition of TdT, the mixture was incubated at 37 °C. The final concentration of each component was as follows: 2 U/µL TdT, 5 µM primer, and 100 µM triphosphates (20 equivalents). The reaction was quenched by the addition of a 5:2:6 mixture of 500 mM EDTA (ethylenediaminetetraacetic acid), a loading solution (30% glycerol, 0.25% bromophenol blue), and 10 M urea. The mixture was immediately heated at 95 °C for 5 min. The products were then analyzed by denaturing PAGE.

### 3.4. Time-Course Analysis

The time-course analysis of the polymerization reaction was performed by initiating the reaction as described in Section 3.3. An aliquot of the reaction mixture was taken for PAGE analysis at defined time points. After quenching the reaction, the samples were stored at −20 °C until the time course was completed. Denaturing PAGE was then carried out and the average extension length was estimated based on the intensity of the bands on the gel. Averages of at least three independent experiments are plotted in the figure.

### 3.5. Electrophoresis Mobility Shift Assay (EMSA)

A FAM-labeled natural DNA primer (5′-FAM-dT_20_-3′) or an artificial ligand-bearing DNA primer (5′-FAM-dT_20_**H**_5_-3′) (5.0 µM) was dissolved in a reaction buffer (20 mM of Tris-acetate (pH 7.9), 50 mM of KOAc) containing various concentrations of MgCl_2_. After the addition of TdT (2 U/µL), the mixture was incubated in the absence of any triphosphate substrate at 37 °C for 1 h. The reaction mixture was immediately analyzed by native PAGE (%T = 4, %C = 3.33) at 10–15 °C using an EDTA-free Tris-borate-NaCl buffer (90 mM of Tris base, 90 mM of boric acid and 50 mM of NaCl). The bands were detected by FAM fluorescence.

## 4. Conclusions

In this study, we investigated the effects of a metal cofactor, Mg^II^ ion, on the TdT-catalyzed polymerization of **H** nucleotides. Enzymatic reactions were analyzed at various concentrations of Mg^II^ ions. At a Mg^II^ concentration as high as 10 mM, the reaction was halted after several **H** nucleotides were appended. In contrast, at lower Mg^II^ concentrations, **H** nucleotides were further appended to the **H**-tailed product to afford longer ligand-bearing DNA strands. EMSA revealed that the binding affinity of TdT to the **H**-tailed DNAs highly depends on the Mg^II^ concentration. While TdT hardly bound to the **H**-tailed DNA strands in the presence of excess Mg^II^ ions (10 mM), TdT accommodated the strands at lower Mg^II^ concentrations (1.0–2.0 mM). This is possibly because the interaction between Mg^II^ ions and the **H**-tailed strands may cause their folding into a certain unfavorable secondary structure to inhibit accommodation by TdT. As a consequence, the further incorporation of **H** nucleotides was impeded.

The enzymatic synthesis of artificial DNAs containing metal-mediated base pairs has recently attracted increasing attention [42,43,44,45,46,47]. While template-dependent DNA polymerases were utilized in these precedent researches, the template-independent terminal deoxynucleotidyl transferase (TdT) was exploited in our study. The discussion presented here emphasizes the importance of the binding affinity of DNA polymerases to the elongated DNA strands, tailed with artificial nucleotides, in the enzymatic synthesis of artificial DNAs. In contrast to typical DNA polymerase, TdT yields single-stranded products, which are flexible enough to fold into unfavorable secondary structures. Thus, the interactions between metal cofactors and ligand-type nucleotides on the single-stranded products should be additionally considered for the TdT-catalyzed polymerization of ligand-bearing nucleotides. Notably, TdT enzyme is expected to more efficiently catalyze the polymerization of other ligand-bearing nucleotides that have lower affinity to Mg^II^ ions, such as mercaptopyridone- and hydroxypyridinethione-bearing nucleotides [48]. Accordingly, the present study will definitely contribute to the future application of metal-mediated base pairs including the construction of DNA-templated metal nanowires and nanomaterials. Further optimization of the TdT-catalyzed synthesis of ligand-bearing artificial DNAs is now underway.

## Abbreviations

**H**	hydroxypydirone-bearing nucleoside
d**H**TP	hydroxypydirone-bearing nucleoside triphosphate
TdT	terminal deoxynucleotidyl transferase
PAGE	polyacrylamide gel electrophoresis
EMSA	electrophoresis mobility shift assay
